# An optimized protocol for single nuclei isolation from clinical biopsies for RNA-seq

**DOI:** 10.1038/s41598-022-14099-9

**Published:** 2022-06-14

**Authors:** Thomas V. Rousselle, Jennifer M. McDaniels, Amol C. Shetty, Elissa Bardhi, Daniel G. Maluf, Valeria R. Mas

**Affiliations:** 1 Department of Surgery, University of Maryland School of Medicine, 670 W Baltimore Street, Baltimore, MD 21201 USA; 2 Institute for Genome Sciences, University of Maryland School of Medicine, Baltimore, MD USA; 3 Program in Transplantation, University of Maryland School of Medicine, Baltimore, MD USA

**Keywords:** Next-generation sequencing, RNA sequencing

## Abstract

Single nuclei RNA sequencing (snRNA-seq) has evolved as a powerful tool to study complex human diseases. Single cell resolution enables the study of novel cell types, biological processes, cell trajectories, and cell–cell signaling pathways. snRNA-seq largely relies on the dissociation of intact nuclei from human tissues. However, the study of complex tissues using small core biopsies presents many technical challenges. Here, an optimized protocol for single nuclei isolation is presented for frozen and RNA*later* preserved human kidney biopsies. The described protocol is fast, low cost, and time effective due to the elimination of cell sorting and ultra-centrifugation. Samples can be processed in 90 min or less. This method is effective for obtaining normal nuclei morphology without signs of structural damage. Using snRNA-seq, 16 distinct kidney cell clusters were recovered from normal and peri-transplant acute kidney injury allograft samples, including immune cell clusters. Quality control measurements demonstrated that these optimizations eliminated cellular debris and allowed for a high yield of high-quality nuclei and RNA for library preparation and sequencing. Cellular disassociation did not induce cellular stress responses, which recapitulated transcriptional patterns associated with standardized methods of nuclei isolation. Future applications of this protocol will allow for thorough investigations of small biobank biopsies, identifying cell-specific injury pathways and driving the discovery of novel diagnostics and therapeutic targets.

## Introduction

The ability to capture gene expression at the single cell resolution has propelled significant discoveries in the biomedical field over the last decade^1,2^. Specifically, single cell RNA-sequencing (scRNA-seq) provides novel insight into cellular heterogeneity across human tissues and facilitates the study of complex diseases. However, an essential prerequisite for scRNA-seq is the processing of tissues into a homogeneous single cell suspension. This process typically relies upon enzymatic incubations at elevated temperatures that can introduce cellular stress artifacts and noise that is only evident after sequencing^3,4^. In addition, with tissues that have been fixed or partially dissociated, the cell isolation method can preferentially select for easily dissociable cell types. This produces a biased representation of cellular architecture^5^. Moreover, human tissue biopsies collected for clinical diagnostics are obtained at any time of day and often away from the laboratory, making the fast processing of fresh viable and healthy cells unpractical. Recently, single nuclei RNA-seq (snRNA-seq) has been introduced as a method to surpass these limitations, whereby cytosolic components are forgone for a clearer snapshot of gene expression^6–8^.

snRNA-seq provides an attractive alternative to overcome technical complications introduced during sample preparation. Nuclei isolation is not biased towards common cell types and is more suited to identify the cellular etiology of pathology, due to morphological similarities. Although snRNA-seq is still within its nascent stages, there is a growing number of publications that have been validated for fresh or flash frozen tissue sections and cell lines. A common strategy utilizes mechanical homogenization in low concentration detergents to disrupt cellular membranes while releasing intact nuclei, followed by removal of aggregated nuclei and debris^9–12^. However, mechanical homogenization leads to over lysis and isolation of unhealthy, ‘blebbing’ nuclei. Aggregate and debris removal is typically accomplished through lengthy ultra-centrifugation or cell sorting techniques which further decrease nuclei quality and total yield. Currently, there are few publications reporting a step-by-step protocol for the application of single nuclei from clinical samples, none of which utilize small needle biopsies^13–15^. There are also no published methods for obtaining intact single nuclei from RNA*later*-stored samples, a common approach used for biobanking clinical biopsies. The limitations of the current methods prevent the processing of such tissues, precluding the evaluation of critical clinical samples collected at unexpected times and away from the laboratory.

Herein, we present an adapted method for single nuclei isolation from small needle biopsies independent of preservation method (e.g., fresh-frozen, flash-frozen, or RNA*later*-stored). The described method excludes lengthy procedures for debris removal. The basic performance of this method was evaluated by viability and total yield using human kidney needle biopsies as well as tissue sections from all major organs from mice. Five human kidneys were isolated and processed through the Chromium Controller platform for snRNA-seq. This method demonstrates proficient single nuclei isolation across multiple samples thereby circumventing the need to optimize multiple protocols.

## Materials

### Reagents

#### Lysis buffer

- 1× PBS pH, 7.4 (Invitrogen, cat. no. AM9625).
- 10 mM Tris–HCL pH 7.5 (Invitrogen, cat. no. 15567027).
- 0.0125% Triton X-100 (Invitrogen, cat. no. A16046.A).
- 1 mM DTT (Thermo Fisher Scientific, cat. no. R0861).
- 0.2 U/μL RNAse inhibitor (Promega, cat. no. PRN2611).

#### Wash and resuspension buffer

- 1× PBS pH, 7.4 (Invitrogen, cat. no. AM9625).
- 2% Bovine serum albumin (molecular grade) (Sigma-Aldrich, cat. no. A9418-50G).
- 0.2 U/μL RNAse inhibitor (Promega, cat. no. PRN2611).

### Equipment

- Bench top centrifuge with swinging bucket rotor (Sorvall, cat. no. 75004240).
- Bench top centrifuge (Sorvall, cat. no.75002447).
- Countess II FL automated cell counter (Thermo Fisher Scientific, cat. no. AMQAX1000).
- Fluorescent dyes: 4ʹ,6-diamidino-2-phenylindole (DAPI) (Invitrogen, cat. no. D1306), ActinGreen (Invitrogen, cat. no. R37110), and Mitotracker CMXros (Invitrogen, cat. no. M7512).
- Micro stir-rod.

## Methods

### Tissue sample collection

Human kidney graft biopsies from five kidney transplant recipients were obtained as part of our ongoing studies following informed consent from all subjects and/or their legal guardian(s). All experimental protocols were approved by the University of Maryland Institutional Review Board (IRB#HP-00092097) and were carried out in accordance with relevant guidelines and regulations. Tissues were collected with 18-gauge biopsy needle and preserved in RNA*later* (Ambion). The samples included normal and disease kidney tissues (one graft biopsy that was characterized as normal/non-specific based on histologically evaluation using Banff scoring^16^) and four samples were characterized as having acute kidney injury (within the first weeks of peri-transplant).

Mouse needle biopsies were collected from heart (left ventricle), liver, kidney, spleen, lung, duodenum, large intestine, bone marrow, and brain (cortex) using a 1.25 mm disposable biopsy needle. Mouse tissues were preserved in RNA*later* and stored in − 80 °C until processing. All experimental methods were approved by the ethics committee established by the Office of Animal Welfare Assurance (OAWA) at the School of Medicine of the University of Maryland to ensure that methods were carried out in accordance with the appropriate approvals and in accordance with the ARRIVE guidelines (IACUC 0920006).

### Single nuclei isolation

Nuclei isolation followed a modified protocol which is described in detail below. Briefly, human kidney needle biopsies were removed from − 80 °C storage and minced to 0.5–1 mm pieces on dry ice with a sterile scalpel blade. The sections were transferred to a 2 mL flat-bottom centrifuge tube containing a micro-stir rod and cold 1 mL lysis buffer. To lyse tissues, samples were placed on a stir plate at 100 RPM and incubated for 5 min while on ice. After lysis, the supernatant was transferred to a 15 mL centrifuge tube containing 6 mL cold wash and resuspension buffer. The remaining tissue sections were washed with an additional 1 mL cold wash and resuspension buffer, and subsequently transferred to the same 15 mL tube placed on ice. To achieve efficient lysis and higher nuclei cell counts, lyses and washes were repeated twice (for a total of three times). An additional 1 mL cold lysis buffer was added to the remaining tissue sample, incubated, transferred, and washed. On the last lysis step, the tissue was incubated for 10 min on ice, stirring at 150 RPM, and processed in the same manner. The 15 mL falcon tube containing extracted nuclei were centrifuged at 600×*g* for 5 min in a 4 °C pre-cooled bucket-rotor centrifuge. The supernatant was discarded, leaving the pellet undisturbed. Using narrow-bore 200 μL gel-loading tips (Thermo Fisher Scientific), the pellet was resuspended in 200 µL cold wash and resuspension buffer and gently pipetted 7–10 times to disrupt aggregated nuclei. The remaining volume was adjusted to 1 mL with cold wash and resuspension buffer. Following, the cell suspension was filtered through a 70 µM FlowMi cell strainer (Sigma-Aldrich) followed by a 40 µM FlowMi cell strainer (Sigma-Aldrich). The effluent was then centrifuged in a 4 °C pre-chilled benchtop centrifuge at 600×*g* for 5 min. The supernatant was discarded, and the pellet resuspended in 200 µL cold wash and resuspension buffer. Isolated nuclei were quantified using DAPI staining solution (Thermo Fisher Scientific) and Trypan blue solution (Thermo Fisher Scientific) while also evaluating cell viability. Nuclei were manually counted using a hemocytometer, and automatically counted using a Countess II FL automated cell counter (Thermo Fisher Scientific) following standard methodology.

#### Tissue homogenization

I. Freshly prepare all reagents on the day of isolation. Autoclave and treat all equipment with RNAsin to reduce contamination.
  - *Critical step* RNAse can greatly reduce nuclei yield and contribute to degradation artifacts within the cDNA. All surfaces and equipment should be decontaminated, and reagents must stay cold throughout the procedure to reduce the activity of RNAses/DNAses. To decrease nuclei retention on plastics, coat pipette tips and centrifuge tubes with 5% BSA to increase nuclei recovery.
II. Prepare the following and place on ice:
  - 2 mL flat-bottom centrifuge tubes equipped with a micro stir-rod also containing 1 mL of lysis buffer.
  - 15 mL centrifuge tubes containing 6 mL wash and resuspension buffer.
III. Remove needle biopsies from − 80 °C freezer storage and place on ice. Section the biopsy into 1 mm pieces using a sterile scalpel blade and place tissue within the 2 mL centrifuge tubes containing a micro stir-rod.
IV. Transfer sample to a magnetic stir plate. Stir at 100 RPM for 5 min on ice.
V. Remove centrifuge tubes from stir plate and allow tissue sections to settle at the bottom. Transfer the supernatant to the 15 mL centrifuge tubes containing 6 mL of wash and resuspension buffer.
VI. Wash the tissue sections with 1 mL of wash and resuspension buffer and transfer supernatant to the 15 mL centrifuge tubes.
VII. Add 1 mL of lysis buffer and stir for an additional 5 min at 100 RPM.
VIII. Repeat steps V–VI.
IX. Add 1 mL lysis buffer and stir for an additional 10 min at 150 RPM.
X. Repeat steps V–VI.
  - *Note* For fibrotic tissues, amend steps VII–X by allowing the tissue to remain in lysis buffer for 20–30 min at 100 RPM. Followed by washing with BSA and proceed with step XI.
XI. Centrifuge the 15 mL tubes at 600×*g* for 5 min in a benchtop centrifuge equipped with a swinging-bucket rotor and precooled to 4 °C.

#### Aggregate and debris removal

XII. Carefully remove the 15 mL tubes from the centrifuge and place on ice. Discard the supernatant and resuspend the nuclei pellet in 200 μL wash and resuspension buffer. Transfer this suspension to a new ice-cold 1.5 mL centrifuge tubes.
XIII. Using 200 μL gel-loading pipette tips, gently pipette the nuclei approximately 10–15 times to disrupt aggregated nuclei.
XIV. Add 800 μL of wash and resuspension buffer to the nuclei suspension and filter solution using a 70 μM FlowMi cell strainer followed by a 40 μM FlowMi cell strainer.
XV. Centrifuge the nuclei suspension in a 4 °C pre-cooled benchtop centrifuge at 600×*g* for 5 min.

#### Nuclei visualization and counting

XVI. Discard the supernatant without disturbing the pellet and resuspend in 200 μL wash and resuspension buffer.
XVII. Using 200 μL gel-loading pipette tips, gently resuspend nuclei.
XVIII. Aliquot 10 μL of the sample solution into a new 0.2 μL microcentrifuge tube and add 90 μL of Bromophenol Blue. Mix well. Aliquot an additional 20 μL of sample solution into a new 0.2 μL tube and add 1 μL of ready-made DAPI solution. Mix well.
XIX. Allow DAPI-stained nuclei to sit on ice for 10 min. While waiting, load the Bromophenol blue stained nuclei onto a hemacytometer. Proceed with nuclei counts using an automated cell counter.
XX. Load 10 μL of the DAPI-stained nuclei onto a hemacytometer for automated cell counting. Record the number of DAPI-positive cells.
XXI. Load the remaining 10 μL of the DAPI-stained nuclei onto a disposable hemacytometer. Visualize and count the nuclei using a fluorescent microscope equipped with 4×/10× objectives.

### Sample processing with the 10× platform

The isolated nuclei were processed through the 10× Chromium workflow per the manufacturer’s instructions. The Chromium Next GEM Single Cell 5ʹ Kit v2 (10× Genomics PN-1000263) followed by the library construction kit (10× Genomics PN-1000190) was used. The resulting libraries were analyzed using the Bioanalyzer 2100 instrument (Agilent) and cDNA quantity was assessed by Qubit fluorometer (Thermo Fisher Scientific). Libraries were then sequenced using a NovaSeq 6000 S4 flow cell (Illumina) targeting 10,000 reads/cell and 100-bp paired-end sequencing.

### snRNA-seq data analysis

The FastQ files generated by the 10× Genomics standard sequencing protocol were aligned to the human pre-mRNA reference sequence (build GRCh38) using CellRanger (10× Genomics, v3) resulting in 3 output files, namely, a table of cell barcodes, a table of gene names, and a gene expression matrix for each individual sample. The CellRanger output was used for preliminary quality control (QC) of the samples. The number of detected features, the depth of gene expression, and the proportion of mitochondrial gene expression in each of the kidney samples were assessed. Genes expressed in few cells (< 3 cells), cells with few expressed genes (< 400 genes), and cells with a high number of expressed genes (> 5000 genes) were excluded based on data-driven cut-offs. Nuclei with > 2.5% of mitochondria gene expression were also removed from downstream analysis. Samples were integrated into one dataset using the R package ‘Seurat’ and tested for any potential batch effects due to technical differences in samples. After correction for possible batch effects, cell clustering using principal component analysis (PCA) followed by uniform manifold approximation and projection (UMAP) was done. Sub-clustering of cell types was based on gene expression of highly variable genes and publicly available knowledge databases to assign potential cell identity to each of the distinct cluster. All statistical analyses and illustrations were generated using R.

## Results

The following optimized protocol describes nuclei isolation compatible with small human and mouse tissue biopsies for 10× Chromium Controller platform and snRNA-seq. These optimizations eliminated cellular debris and allowed for a high yield of high-quality nuclei and RNA for library preparation and sequencing. The procedure is gentle, fast, and efficient using readily available laboratory supplies.

**Figure 1:**
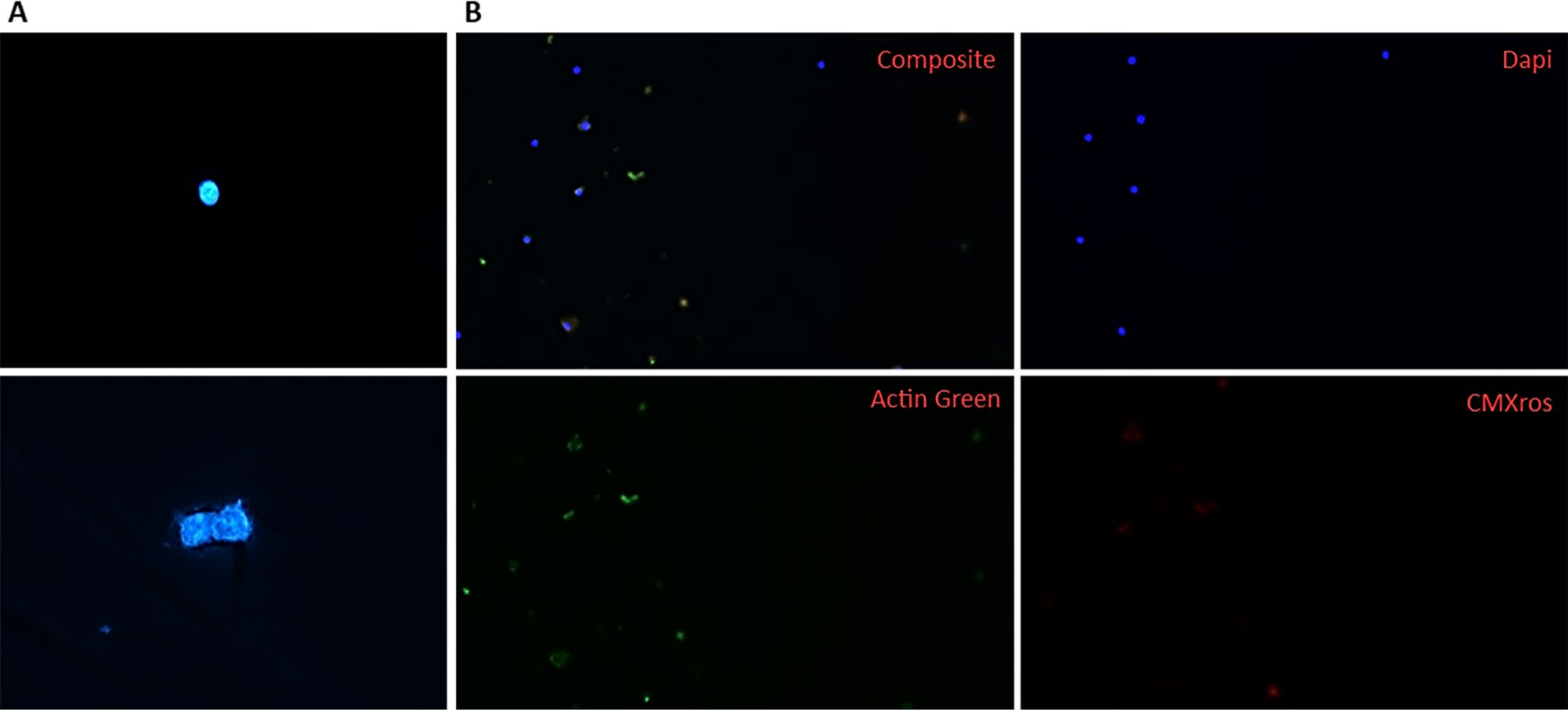
Examination of nuclei morphology by fluorescent staining. (**A**) Representative images of DAPI-stained nuclei showing successful isolation of a healthy single nucleus (top) and nuclei aggregation (bottom). Nuclei doublet also shows abnormal nuclear morphology. (**B**) Positive fluorescence staining with DAPI, CMXros, and Actin Green (clockwise). The composite image (top left) confirms live cell staining after enriching for nuclei using the protocol.

**Figure 2:**
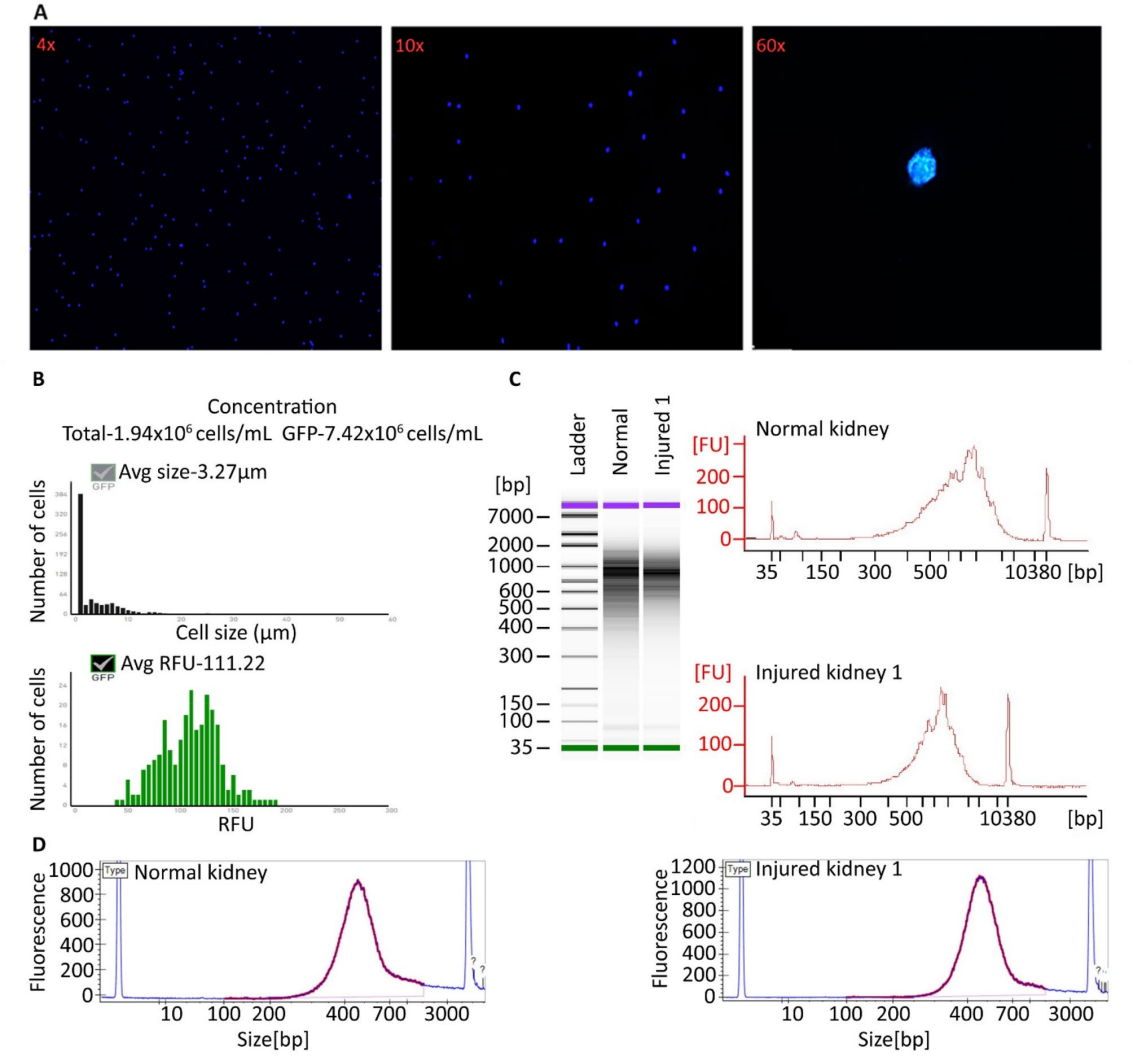
Quality control assessment of nuclei isolation from human kidney biopsies. (**A**) Representative images for morphological analysis of DAPI-stained nuclei. Magnification is in increasing order. (**B**) Cell number acquired by Countess II automated cell counter represents the total number of nuclei (GFP) and background noise (cellular debris, total). (**C**) Agilent bioanalyzer analysis of the cDNA traces. Fragments are expected to be > 600 bp with a max length of 2000 bp. Top, normal sample; bottom, injured kidney 1 sample. (**D**) Agilent bioanalyzer of post-library preparation quality. A size range of 400–600 bp is expected.

**Figure 3:**
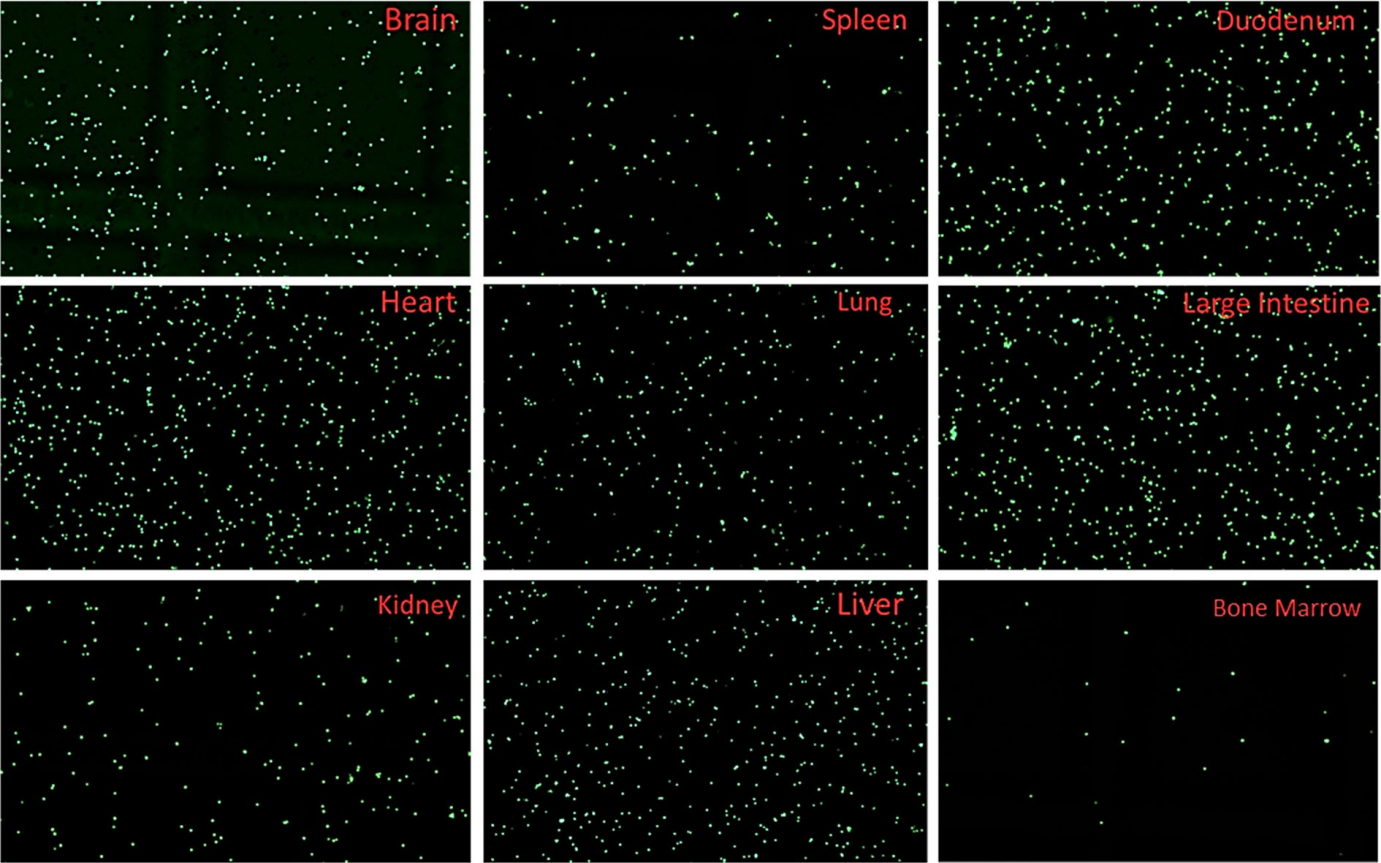
Representative images of single nuclei isolation from mouse tissues. Tissues were collected using needle biopsies and imaged at × 4 magnification. Green, nuclei.

Five human kidney grafts were independently processed, minced, and transferred to a gentle lysis buffer with agitation, allowing for physical dissociation of cell membranes. During this step, samples are to remain in full contact with lysis buffer containing RNase inhibitors and to remain on ice. Sequential lysis and washes increased the final number of collected nuclei and reduced the presence of contaminants. In line with standard protocols, the centrifugation steps were done at low speed to minimize nuclei damage. Cellular debris was removed by passing the sample through a 70 µm filter followed by a 40 μm filter. An additional step involving resuspension of the nuclei with a narrow-bore gel loading pipette tip is included to reduce nuclei multiplets or nuclei aggregation (Fig. 1), a common isolation obstacle leading to sample reduction. Note, excessive pipetting can lead to premature lysis. Released intact nuclei were stained with DAPI, ActinGreen, and Mitotracker CMXros (Fig. 1) to evaluate nuclei morphology, actin cytoskeleton contamination, and mitochondrial contribution, respectively. Nuclei blebbing was not observed (Fig. 2A). Nuclei were then counted using a hematocytometer and a Countess electronic cell counter (Fig. 2B). To show the applicability, feasibility, and reproducibility of the method, the protocol was repeated using needle biopsies collected from various mouse tissues without proceeding through the 10× Chromium Controller platform. Specifically, to evaluate the performance of the method independently of tissue type, multiple tissues from mice were used for nuclei isolation. Nuclei counts and integrity were consistent with human kidney isolations (Fig. 3). Fluorescent staining also confirmed that nuclei were indeed viable at the end of the protocol with little cellular membrane contamination. Depending on the number of samples and sample complexity, this procedure can be completed in 90 min or less for immediate processing through the 10× Chromium Controller platform.

Isolated nuclei from human kidney biopsies were further examined. The manufacturer’s protocol for nuclei lysis and cDNA generation (10× Genomics: user guide CG000331, Rev C) was followed as recommended. On average, 2483 (N = 13,075 total) nuclei were isolated across normal and acute injury (Supplementary Table 1). Quality control metrics verified the isolation of high-quality nuclei. Bioanalyzer analysis of cDNA traces pre-library (Fig. 2C) and post-library (Fig. 2D) preparation yielded the expected fragment size and peaks. A range of 1–48 ng of total cDNA was used to generate libraries (Supplementary Table 1). An input of 1 ng is the lowest concentration suggested by 10× Genomics, producing high library quality without traces of degradation (Fig. 2D).

**Figure 4:**
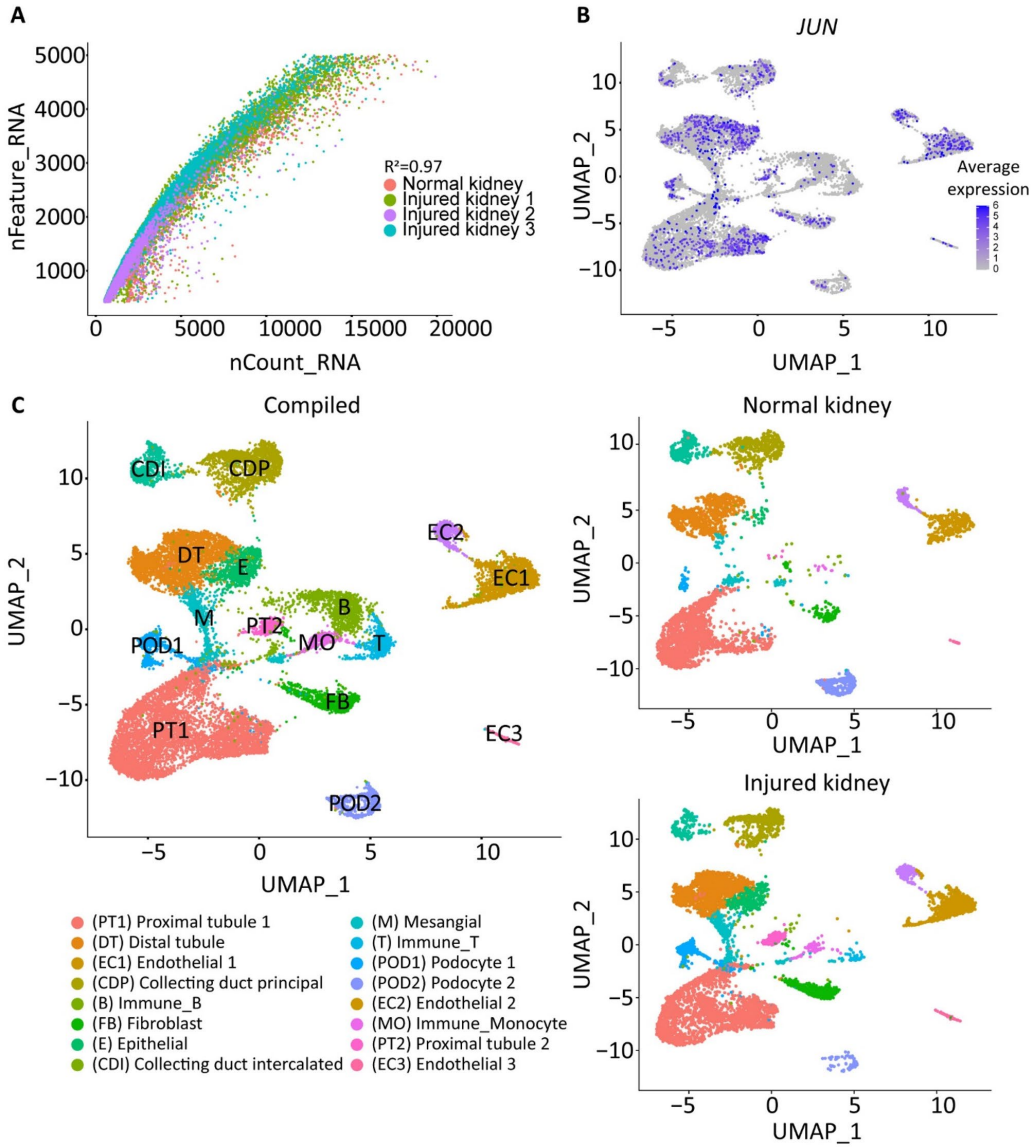
Assessment of single nuclei quality after snRNA-seq. (**A**) Dot plot representation of the relationship of the number of genes detected (y-axis) against the total number of RNA reads (x-axis). R^2^ = 0.97. (**B**) Average expression of *JUN* stress response pathway across each cell population. (**C**) (left) UMAP visualization of a total of 13,075 nuclei partitioned into 16 clusters from a total of 5 human kidneys after quality control filtering. Each cluster is labeled by color. (right) Individual UMAP visualization of the normal and injured kidney samples.

Over 880 million reads were sequenced (Supplementary Table 2). The Q30 scores of barcodes, RNA reads, and unique molecular identifiers (UMIs) were all above 84% (Supplementary Table 2). Data was normalized by expression values across cells, dividing counts for each gene by total counts in the cell. The number of UMIs were significantly correlated with the amount of mRNA for all samples (R^2^ = 0.97) (Fig. 4A). Mitochondria-associated genes were identified as a minor contamination contributor (R^2^ = 0.17) (data not shown). Further, to examine the effect of the described tissue disassociation method on isolated nuclei, the JUN stress pathway expression was quantified (Fig. 4B). The relative expression of *JUN* across samples was low.

Sample quality was also determined by the ability to define cell types using unsupervised clustering of transcriptional data. Each sample processed using the described protocol gave rise to 16 clusters that included both common and rare kidney cell types (Fig. 4C), suggesting that despite sample complexity this protocol was able to obtain high quality transcriptomes of many unique cell types. These include proximal tubules (PT), distal tubules (DT), collecting ducts (CD), endothelial cells (EC), epithelial cells (E), podocytes (POD), mesangial cells (M), fibroblasts (F), T cells (T), and monocytes (MO) (Fig. 4C). Tubule clusters (proximal (PT) and distal (DT)) comprised the most prevalent cell types. All clusters were detected in each group; however, significant cluster heterogeneity was observed between groups (Fig. 4C).

**Figure 5:**
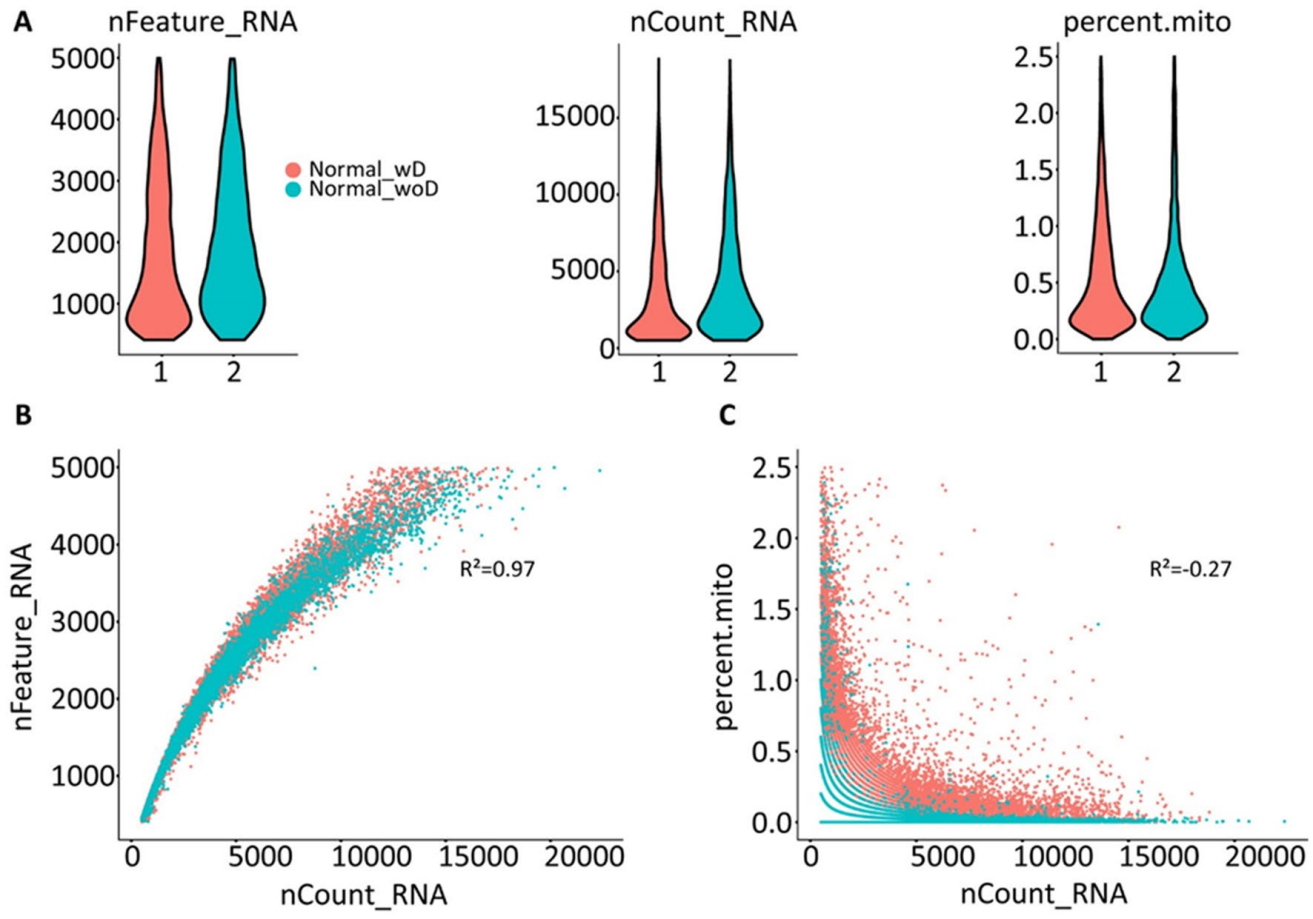
Comparative sequencing performance metrics for single nuclei isolation by traditional methods. Sequencing quality control metrics comparing a representative normal sample isolated (**A**) using douncing (1) and our described method without douncing (2). Dot plot representation of each group depicting the relationship of the (**B**) number of genes detected (y-axis) against the total number of RNA reads (x-axis) (R^2^ = 0.97) and (**C**) percent of mitochondria reads (y-axis) against the total number of RNA reads (x-axis) (R^2^ =  − 0.27).

To rigorously evaluate the performance of the described protocol compared to the standardized protocol described by Kirita et al.^17^, we examined sequencing quality and stress response activation between the two datasets. All samples were cryopreserved in RNA*later*, eliminating sample storage variation. Herein, we used labels “w_douncing” and “wo_douncing” to refer to nuclei isolated using standardized and our described method, respectively. No significant differences were observed in the number of detected features, the depth of gene expression, and the proportion of mitochondrial gene expression (Fig. 5A). The summaries for detected mitochondria genes and unique genes in the pool of total RNA reads are shown in Fig. 5B,C. The vast majority of reads derived from both methods overlapped in quality control metrics following the same trend for correlation. The number of reads were positively correlated with the amount of mRNA for all the samples (R^2^ = 0.97). Mitochondrial content of < 2.5% with relatively low mitochondrial gene expression (R^2^ =  − 0.27; Fig. 5C), showing that the total number of RNA reads were not significantly correlated with the percentage of mitochondrial genes. Stringent quality control metrics yielded high confidence in the data generated from both methods.

**Figure 6:**
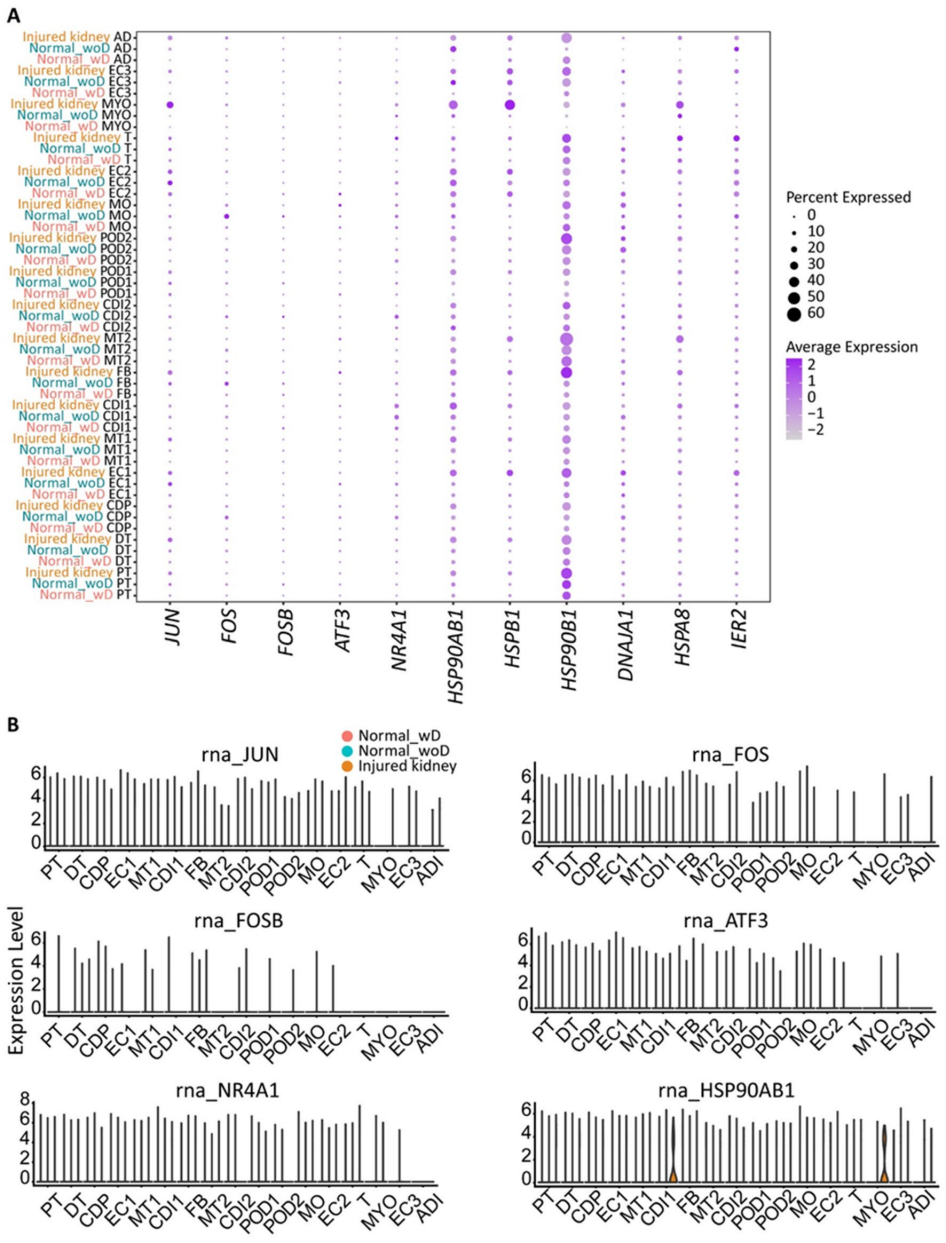
Analysis of cellular stress response. (**A**) Gene expression of stress response markers from isolated kidneys with (Normal_wD) and without douncing (Normal_woD and Injured kidney). Kidneys without douncing were isolated following our described protocol. The size of the dot represents the percentage of cells in the corresponding cluster expressing the marker and the color of the dot represents the average gene expression value. (**B**) RNA expression levels by cluster. Cell clusters: *ADI* adipose cell, *CDI1* colleting duct intercalated, *CD12* collecting duct intercalated 2, *CDP* collecting duct principal, *DT* distal tubule, *EC1* endothelial cell 1, *EC2* endothelial cell 2, *EC3* endothelial cell 3, *FB* fibroblast, *MO* monocyte, *MT1* mixed tubule 1, *MT2*, mixed tubule 2, *MYO* myocyte, *POD1* podocyte 1, *POD2* podocyte 2, *PT* proximal tubule, *T* T cell.

As previously mentioned, activation of the stress response by cellular disassociation is a major hurdle in single nuclei studies. We examined the expression levels of a set of canonical stress-related genes including *ATF3*, *DNAJA1*, *FOS*, *HSPA8*, *HSP90AB1*, *HSPB1*, *HSP90B1*, *IER2*, and *NR4A1* in addition to *JUN* expression (Fig. 6A,B). To further validate minimal activation of the cellular stress response, gene expression levels were compared according to their isolation method (w_ and wo_ douncing) and kidney sample classification type (normal and injured). From the evaluations, there were minimal variation of stress-related gene expression and present in less than 20% of total cells (Fig. 6A). However, for samples pathologically characterized with acute kidney injury labeled as injured kidney, *JUN* expression is prominent in the proximal tubule cluster (Fig. 6A). This finding is expected since tubulointerstitial damage was observed. Further analysis of individual RNA transcript expression per individual cell cluster validated that cellular stress response was not activated related to the isolation method (Fig. 6B). Importantly, the gene expression levels of *HSP90AB1* and *HSP90B1* were upregulated in the injured kidney and downregulated in the transcriptome of the normal kidney isolated using both methods. With this result, we can strongly conclude that we are able to detect cellular stress in injured kidneys, but these pathways are not induced in the normal kidney. Our results indicate that our described protocol does not introduce cellular stress related to tissue disassociation that may influence biological interpretations following snRNA-seq.

## Discussion

Single nuclei profiling is a powerful tool in understanding cell-specific gene expression profiles, cellular crosstalk, and molecular dynamics over time. snRNA-seq reduces the influence of dissociation bias and stress-induced transcriptional artifacts compared to scRNA-seq, allowing for better representation of native cell states^6^. Despite its advantages, there are several technical challenges impeding the isolation of quality nuclei in concentrations high enough for downstream analysis^18,19^. A low number of cells results in a reduction of assay sensitivity and confidence of gene expression analysis. This described protocol offers an alternative isolation method that is time-effective and reduces excessive manipulation yielding high-quality transcriptomics data. Traditional isolation methods are labor intensive and costly, involving sample homogenization (usually performed with dounce homogenizers or beads), cell sorting, and ultra-centrifugation, all of which have been omitted within the modified protocol. The use of readily available laboratory supplies allows for the broad application of single nuclei technology in many different laboratory settings^20^.

To verify the effectiveness of the protocol for snRNA-seq, both human and mouse tissue biopsies were assessed. These tissues were preserved with RNA*later* or fresh frozen. Intact nuclei evidenced by normal morphology were consistently observed for each sample, indicating no technical variation from preservation method or sample variability across tissue types. The accuracy of the method was confirmed by UMAP analysis. Based on the results, each of the 5 kidney graft samples, including those with peri-transplant acute kidney injury, exhibited 16 shared cell clusters. This demonstrates the strength of the proposed approach. Isolation of thousands of nuclei with little manipulation generated reproducible kidney cell clusters. Thus, this protocol can be safely used to provide high quality data about normal and diseased transcriptomes at single cell resolution.

The lower number of aligned sequences does not necessarily reflect the performance of the protocol but the cellular diversity in complex tissues as normal and diseased samples were included in this study. Nonetheless, this did not hinder the detection of cells that are present in low proportions in the kidney (e.g., immune cells, podocytes) or common cell types (e.g., epithelial proximal tubular cells, endothelial cells) (Fig. 4C). The low detection rate of immune cells has been commonly associated with snRNA-seq compared to scRNA-seq^6,21^. However, the described protocol overcomes this challenge, allowing for the recovery of at least 3 independent immune cell types (e.g., B cells, T cells, and monocytes) from a total of 2296 immune cells (Fig. 4C).

The ability to detect a heterogenous population of immune cells was directly compared to previously reported findings on snRNA-seq isolated from complex tissues. The current protocol performance was consistent or better than other studies in terms of immune cell abundance and the number of immune cell clusters identified^6,22,23^. Despite differences in tissue origin (mouse *vs.* human tissues), most studies identified 1–2 clusters with a high abundance of monocytes or macrophages^6,22,23^. One study has also cited that low cell proportion and improper maintenance of an optimal temperature are factors for loss of immune cell populations^22^. To minimize loss of immune cells, samples and materials from the current study remained on ice and were immediately processed through the 10× Chromium pipeline. Samples exhibited limited activation of the stress response pathway, which may also aid in immune cell recovery (Fig. 6A,B). Expectedly, the proximal tubule cluster within the injured kidneys exhibited the highest level of *JUN* expression as compared to normal kidneys, likely due to an upregulated response to the sustained acute injury^24,25^. This protocol can be used to capture a diverse set of cells from complex tissues to further elucidate mechanisms of human disease. Such methods allow for the study of transcriptomic profiles and cell-to-cell interaction networks that have been missed by previous techniques. Isolating immune cells is essential for the study of poorly understood renal diseases (e.g., glomerular diseases) and is essential for understanding the activation patterns, maladaptive processes, and recruitment of immune cells in the kidney (e.g., kidney transplantation).

The described protocol has been shown to achieve high quality sequencing data starting from a low or high number of nuclei ranging from > 2000 to ≤ 10,000 nuclei. Low input depends on the complexity of tissue and the user’s adherence to the protocol. Low input has preserved transcriptional signatures and clustering while also eliminated variance across samples. Thus, this protocol enables the study of homogeneous and heterogeneous samples to define a disease phenotype independent of starting material. This method is applicable to both low and high nuclei numbers generating high quality sequencing samples to study small needle biopsies at single cell resolution.

Likewise, the range of mapped and unmapped reads depends widely on the amount of starting RNA and artificial product sizes generated during amplification^26^. The number of genes ranged from 21,165–26,742, indicating moderate-to-high library complexity. The number of genes detected is congruent with results of earlier studies^6,27^.

Overall, the proposed methods and previously published protocols illustrate similar performance despite small tissue size obtained from core biopsies. For example, after quality control metrics, an average of 1082 unique transcripts per nucleus were identified. Other studies have comparable results using the 10× Chromium platform^15^.

Despite the demonstrated success, some limitations are presented. Even with minor sample manipulation, nuclei loss is still detected. cDNA amplification is a major step in sample preparation. A lower concentration of cDNA was observed after 12 amplification cycles (10× Genomics: user guide CG000331, Rev C), potentially accounting for a lower number of detected genes. Due to the lower amount of RNA derived from the nucleus, the amplification cycle can be increased. This change can affect the number of genes detected and introduce amplification bias.

In conclusion, this protocol provides an alternative approach for nuclei enrichment from small core needle tissue biopsies. This protocol has been proven successful in the isolation of clinical samples, including but not limited to, human kidneys as well as the major organs from mice. The application of the described technique supports the advancement of single nuclei transcriptomics studies in which high nuclei counts can be achieved with limited cellular contamination. Without compromising sequencing or nuclei quality, our protocol performs as well as currently available methods. The investigation of kidneys and other organs at a single-nuclei resolution has the potential to revolutionize our ability to study human disease and improve future targeted therapeutic strategies.

### Supplementary Information

Supplementary Tables.

## Data Availability

The raw sequence data generated in this study were deposited in the Gene Expression Omnibus (GEO) under the accession number GSE195719. The data can be accessed by visiting https://www.ncbi.nlm.nih.gov/geo/query/acc.cgi?acc=GSE195719.
