# Author Correction: An optimized protocol for single nuclei isolation from clinical biopsies for RNA‑seq

**DOI:** 10.1038/s41598-024-65796-6

**Published:** 2024-07-04

**Authors:** Thomas V. Rousselle, Jennifer M. McDaniels, Amol C. Shetty, Elissa Bardhi, Daniel G. Maluf, Valeria R. Mas

**Affiliations:** 1 Department of Surgery, University of Maryland School of Medicine, 670 W Baltimore Street, Baltimore, MD 21201 USA; 2 Institute for Genome Sciences, University of Maryland School of Medicine, Baltimore, MD USA; 3 Program in Transplantation, University of Maryland School of Medicine, Baltimore, MD USA

Correction to: *Scientific Reports* 10.1038/s41598-022-14099-9, published online 14 June 2022

The original version of this Article contained a repeated error in the Materials section, under the subheadings ‘Lysis buffer’ and ‘Wash and resuspension buffer’, where the RNase inhibitor unit was incorrect.

As a result,

“0.2 U/mL RNAse inhibitor (Promega, cat. no. PRN2611).”

now reads:

“0.2 U/μL RNAse inhibitor (Promega, cat. no. PRN2611).”

The original Article has been corrected.

