# Supplementary material for: An optimized protocol for single nuclei isolation from clinical biopsies for RNA-seq
Source: Sci Rep. 2022 Jun 14;12:9851. doi: 10.1038/s41598-022-14099-9 (PMC9198012; doi:10.1038/s41598-022-14099-9)
Supplement: Supplementary file 1 — Supplementary Tables. [file 41598_2022_14099_MOESM1_ESM.docx]

**Supplementary Table 1. Summary of pre- and post-library preparation**

| Sample  ID | Pre-library | | Post-library | | |
| --- | --- | --- | --- | --- | --- |
|  | cDNA conc.  (ng/µl) | Total cDNA conc.  (ng) | cDNA con.   (ng/μl) | Total cDNA conc.  (ng) | Avg library size  (bp) |
| Normal | 2.42 | 108.8 | 1.20 | 42.00 | 528 |
| Injured kidney 1 | 0.39 | 17.55 | 1.26 | 44.10 | 485 |
| Injured kidney 2 | 1.33 | 59.85 | 1.42 | 49.70 | 505 |
| Injured kidney 3 | 0.97 | 43.65 | 1.56 | 54.60 | 500 |
| Injured kidney 4 | 0.19 | 8.550 | 1.22 | 42.70 | 436 |
| Average | 1.06 | 47.680 | 1.332 | 46.620 | 490.8 |

|  |  |  |  |  |  |
| --- | --- | --- | --- | --- | --- |

**Supplementary Table 2. Summary of 10x cell ranger results**

| Sample ID | Estimated number of cells | Total genes detected | Mean reads per cell | Total number of reads | Valid UMIs (%) | Q30 in barcode (%) | Q30 in RNA reads (%) | Q30 in UMI (%) |
| --- | --- | --- | --- | --- | --- | --- | --- | --- |
| Normal | 2,846 | 25,411 | 399,722 | 1,137,607,496 | 99.6 | 94.0 | 83.5 | 94.1 |
| Injured kidney 1 | 1,404 | 24,720 | 518,875 | 728,500,653 | 99.7 | 95.1 | 82.2 | 94.4 |
| Injured kidney 2 | 4,513 | 26,742 | 194,998 | 880,026,080 | 99.8 | 95.0 | 86.9 | 94.3 |
| Injured kidney 3 | 3,143 | 25,638 | 228,749 | 718,959,240 | 99.7 | 94.4 | 84.9 | 93.9 |
| Injured kidney 4 | 1,169 | 21,165 | 841,156 | 938,311,909 | 99.7 | 94.6 | 83.1 | 94.1 |
| Average | 2,483 | 24,735 | 436,700 | 880,681,076 | 99.7 | 94.6 | 84.1 | 94.2 |
